# Phosphatidylserine eversion regulated by phospholipid scramblase activated by TGF-β1/Smad signaling in the early stage of kidney stone formation

**DOI:** 10.1007/s00240-021-01292-0

**Published:** 2021-12-03

**Authors:** Xiu Guo Gan, Hai Tao Xu, Zhi Hao Wang

**Affiliations:** Department of Urology, First Affiliated Hospital of Harbin Medical University, Harbin Medical University, Harbin, China

**Keywords:** Phospholipid scramblase, Phosphatidylserine, Kidney stone, TGF-β1

## Abstract

The mechanism underlying phosphatidylserine eversion in renal tubule cells following calcium oxalate-mediated damage remains unclear; therefore, we investigated the effects of TGF-β1/Smad signaling on phosphatidylserine eversion in the renal tubule cell membrane during the early stage of kidney stone development. In a rat model of early stage of calcium oxalate stone formation, phosphatidylserine eversion on the renal tubular cell membrane was detected by flow cytometry, and the expression of TGF-β1 (transforming growth factor-β1), Smad7, and phospholipid scramblase in the renal tubular cell membrane was measured by western blotting. We observed that the TGF-β1/Smad signaling pathway increased phosphatidylserine eversion at the organism level. The results of in vitro studies demonstrated that oxalate exposure to renal tubule cells induced TGF-β1 expression, increasing phospholipid scramblase activity and phosphatidylserine eversion in the renal tubule cell membrane. These results indicate that TGF-β1 stimulates phosphatidylserine eversion by increasing the phospholipid scramblase activity in the renal tubule cell membrane during the early stage of kidney stone development. The results of this study form a basis for further detailed research on the development of therapeutic agents that specifically treat urolithiasis and exert fewer adverse effects.

## Introduction

Urolithiasis is one of the most common diseases affecting humans and is associated with multiple urological complications [1]. Calcium oxalate(CaOx) reportedly injures cultured renal tubular cells, after which cell membrane phosphatidylserine (PS) flips from the inner layer to the outer layer in vitro to play important roles in kidney stone formation [2]. Similar findings have been reported by various studies [3–6] conducted at the cellular level. However, the mechanism underlying PS externalization in renal tubule cells caused by CaOx-mediated damage remains unclear. Previous studies have showed that a high concentration of calcium oxalate leads to the overproduction of reactive oxygen species (ROS) [7], which subsequently contributes to PS externalization in renal tubule cells to result in nucleation and growth of CaOx crystals [8]. ROS and lipid peroxidation strongly activate phospholipid scramblase (PLSCR) [9], whereas exposure to ROS scavengers, such as glutathione, coenzyme Q10, or idebenone (a synthetic coenzyme Q10 homolog), reduces the activation of PLSCR in polycystic kidney disease [10]. PLSCR, which is anchored to the cell membrane [11], functions as one of the three phospholipid-flipping enzymes capable of catalyzing the rapid two-way movement of PS on both sides of the membrane along a concentration gradient [12]. Under physiological conditions, the cell membrane is in an asymmetric state, and PLSCR has no effect on the membrane [13]. However, following injury of eukaryotic cells, PLSCR is activated and moves PS from the inner to the outer layer [14, 15]. Additionally, overexpression of PLSCR in Chinese hamster ovary K1 cells reportedly stimulates PS externalization, enhancing outward movement of PS to the cell surface during apoptosis [16]. Therefore, we hypothesized that PLSCR is also involved in PS externalization in the renal tubule cell membrane via activation of ROS.

A potential molecular mechanism of oxalate-induced ROS production occurs through induction of TGF-β1/Smad signaling, which may play a role in the production of ROS [17, 18]. Indeed TGF-β1/Smad signaling reportedly is involved a myriad of kidney diseases, such as glomerulonephritis, renal interstitial fibrosis, and nephrolithiasis [19]. Moreover, mononuclear cells treated with TGF-β1 exhibit PS externalization in the cell membrane by causing the flipping of PS from the inner to the outer layer [20]. However, to our knowledge, the relationship between TGF-β1/Smad signaling and PS eversion in renal tubule cell membranes remains unknown. We hypothesized that TGF-β1 participates in PS eversion in renal tubule cell membranes by activating PLSCR in the early stage of rat kidney stone formation mediated by ROS.

Therefore, we investigated whether TGF-β1/Smad signaling stimulates PS externalization through ROS activation and whether TGF activates PLSCR in the renal tubule cell membrane during the early stage of kidney stone development in vitro and in vivo, thereby laying a theoretical foundation for the etiology of kidney stone formation.

## Materials and methods

### Rat model of early stage CaOx kidney stone formation

Sixty clean 3-month-old male Wistar rats weighing 200 ± 20 g were provided by the Experimental Animal Center of Harbin Medical University and housed at room temperature (22 °C ± 2 °C) and 50–70% humidity. After 3 days of adaptive breeding, they were randomly divided into three groups (20 rats/group)—control, kidney stone, and kidney stone + SB431542 (a TGF-β1/Smad signaling pathway inhibitor).

Kidney stone formation is initiated as an early pathological change after renal tubular cell injury [21, 22]. To study this process of injury, a rat model of early stage kidney stone formation was established, as a standardized approach has not yet been reported. To observe the early changes after renal tubular cell injury, we applied the method reported by Li et al. [23]. Briefly, the rats in the control group were administered 2 mL of distilled water daily via oral gavage, whereas the rats in the kidney stone group were administered 2 mL of 1% ethylene glycol solution and 1% NH_4_Cl solution daily via oral gavage. The rats in the kidney stone + SB431542 group were intraperitoneally injected with SB431542 co-solvent (5 mg/kg, S1067; Selleckchem, Shanghai, China) every 7 days [24]. Food and water were provided ad libitum. On day 14, the rats were euthanized by anesthetic overdose (pentobarbital sodium, 200 mg/kg, via intraperitoneal injection) and their kidneys were excised. The left kidneys were preserved immediately in liquid nitrogen for the subsequent protein detection. The right kidneys were fixed, and then dehydrated and embedded in paraffin wax. All animal studies were conducted according to the National Institutes of Health Guide for the Care and Use of Laboratory Animals. The experimental procedures were approved by the Animal Experiment Use Ethics Committee of the First Affiliated Hospital of Harbin Medical University (Approval number: 2020005, Harbin, China).

### Crystal formation in renal tissue

The tissue samples were stained using Pizzolato method [23] and CaOx crystals were observe under a polarized light optical microscope (Sinico Optical Instrument Co., Shenzhen, China). Pizzolato-positive regions were measured and expressed as permillages of the total tissue area of cross-sections using ImageJ 1.49v (National Institutes of Health, USA).

### Detection of PS in renal tubular cell membrane in vivo by flow cytometry

Renal epithelial cell PS exposure was evaluated using a fluorescein isothiocyanate (FITC)-labeled Annexin V staining assay, as previously described [2]. The cells were observed (in each group) under a confocal laser microscope (Olympus, Tokyo, Japan). The amount of PS externalization was determined as the percentage of Annexin V-positive cells. Annexin V fluorescence of the samples was measured using a flow cytometer (488 nm excitation/530 nm emission; Beckman Coulter, Brea, CA, USA).

### Measurement of TGF-β1 and Smad7 levels in renal tubular cell membrane by western blotting

After recovery of the frozen tissue samples, protein was extracted using the natural membrane protein extraction kit ProteoExtract (M-PEK) (MERCK Co., Kenilworth, NJ, USA), and the protein concentration was determined using a Bio-Rad protein assay kit (Hercules, CA, USA) according to manufacturer’s instructions. Subsequently, 40 μg of protein was loaded on an 8% SDS–polyacrylamide gel (stacking gel 100 V, resolving gel 200 V) for 1 h at an electrode constant current of 300 mA. The proteins in the gel were transferred onto a nitrocellulose membrane (Kexing Co., Beijing, China), blocked for 1 h at 37 °C in 5% skim milk powder, and incubated overnight at 4 °C with sheep anti-rat TGFβ1 (ab208466; Abcam, Cambridge, UK) and Smad7 (66,478; Proteintech, Wuhan,China) antibodies [25]. Horseradish peroxidase-labeled rabbit anti-sheep secondary antibody was added (1:400; Nakasugi Jinqiao Co., Beijing, China) and incubated at 37 °C for 1 h. The signal was detected using the enhanced chemiluminescence reagent (Abcam). Positive bands were analyzed using Gel-Pro 4.0 gel optical density analysis software, and the cumulative optical density (IOD value) was measured, with the R value representing the relative protein content as follows: R = reference IOD value of PLSCR (target)/reference IOD value of GAPDH (reference protein).

### Cell culture and RNA interference

A renal epithelial cell line (MDCK cells, CCL34, passages 53–90; ATCC, Manassas, VA, USA) was grown in Dulbecco’s modified Eagle medium supplemented with antibiotics (100 U/mL penicillin, 100 mg/mL streptomycin; Life Technologies, Carlsbad, CA, USA) and 10% fetal bovine serum (Life Technologies Co.) at 37 °C in a 5% CO_2_ atmosphere and subcultured with 0.25% trypsin and 1 mM ethylenediaminetetraacetic acid (Life Technologies Co., CA, USA). The cells were treated with CaOx (0.5 mM; Sigma-Aldrich, St. Louis, MO, USA) for 2 h and with 0.5 mM CaOx and 100 μg/mL neutralizing anti-TGF-β1 antibody (R&D Systems, Minneapolis, MN, USA) for 2 h. All experiments were performed in triplicate.

To determine the role of PLSCR in PS eversion, renal tubular epithelial cells were transfected with PLSCR siRNA [26]. siRNA was designed and synthesized by Anhui General Biological System (Anhui, China; see Table 1). Lipofectamine™ 2000 (Sunshine Biotechnology Co., Ltd., Shanghai, China) was used for transfection according to the manufacturer’s instructions. After 24 h of transfection, the cells were digested with trypsin, centrifuged at 1000×*g* for 5 min, washed twice with PBS, and centrifuged again. The supernatant was discarded, and the pellet was suspended in PBS to form a single-cell suspension of density 1 × 10^6^ cells/mL. The positive rate of FAM (carboxyfluorescein) expression was 93% ± 5% as determined by flow cytometry. To further assess the relationship between TGFβ1 and PLSCR, the cells were treated with 0.5 mM CaOx or 10 ng/mL TGF-β1 for 2 h, and subsequently transfected with PLSCR siRNA.

**Table 1:** siRNA sequences for *PLSCR*

siRNA	Sequence (5′-3′)
si-NC	UUCUCCGAACGUGUCACGUTT
siRNA-390	UGGACAAACAAAACUCACATT
siRNA-85	GGGCCAUCUAGACCUUUUATT
siRNA-911	GGAGAGACCACUAAGAUGUTT

### Measurement of PLSCR level in renal tubular cell membranes by western blotting

Protein was extracted using a natural membrane protein extraction kit ProteoExtract (Chemical Book Co., Beijing, China), and the protein concentration was determined using a Bio-Rad protein assay kit (Noble-Ryder Co., Beijing, China). Subsequently, 40 μg of protein was loaded on an 8% SDS–polyacrylamide gel (stacking gel 100 V, resolving gel 200 V) for 1 h at a constant current of 300 mA. Proteins in the gel were transferred onto a nitrocellulose membrane (Kexing Co., Shenzhen, China) and blocked with 5% skim milk for 1 h at 37 °C and incubated overnight at 4 °C with sheep anti-rat PLSCR monoclonal antibody (1:200, PAB781Hu01; Abcam, Guangzhou, China). Horseradish peroxidase-labeled rabbit anti-sheep secondary antibody was then added (1:400) and the mixture was incubated at 37 °C for 1 h. After washing the membrane, the signal was detected with enhanced chemiluminescence reagent (Abcam, Guangzhou, China). Positive bands were analyzed using Gel-Pro 4.0 gel optical density analysis software, and the IOD value was measured, with the R value representing the relative protein content as described above.

### Measurement of membrane phosphatidylserine distribution

The amount of cell membrane PS eversion was determined using the fluorescence quenching method described by Kim et al. [27] and our research team [2], with nitrobenz-2-oxa-1,3-diazole (NBD, Shanghai, China) as the fluorescence probe. After labeling the PS in the outer layer of the cell membrane with NBD, the cells were cultured in PBS containing 5 mM diisopropylfluorophosphate. Thereafter, PBS containing calcium oxalate (1.0 mM) was added and the cells were incubated at 37 °C. An equal amount of cell suspension was discharged every 10 min, and the fluorescence intensity was recorded using an RF-5000 spectrofluorometer (Shimadzu, Kyoto, Japan). The settings of the spectrofluorometer were as follows: 10 nm slit width for an emission wavelength of 530 nm; and 5 nm slit width for an excitation wavelength of 470 nm. Next, the average fluorescence value (FT) was recorded over 50 s. The average reduced fluorescence intensity (FD) was also recorded after using a dithionite solution to quench the NBD fluorescence from the outer layer of the cell. Thereafter, 1% (w/v) Triton X-100 was added to permeabilize the cell membrane. The NBD fluorescence from the inner layer of the cell membrane was then quenched, and the fluorescence intensity (F0) was recorded. The percentage of NBD-PS in the inner lobules was later calculated using the following equation: percentage of internalized NBD-PS = 100 × [(FD − F0)/(FT − F0)].

The percentage of PS externalization was determined according to the principle used for the measurement of NBD-PS internalization. The percentage of NBD-PS in the outer lobules was also calculated using the following equation: percentage of NBD-PS in outer lobules = 100 × [(FT − FD)/(FT − F0)].

### Statistical analysis

Continuous variables are expressed as mean ± standard deviation. A non-parametric paired rank-sum test, *t*-test, and chi-square test were used. All analyses were performed using SPSS software 19.0 (SPSS, Inc., Chicago, IL, USA). Results with *P* < 0.05 were considered statistically significant.

## Results

### TGF-β1/Smad signaling stimulates PS externalization in vivo

#### Crystal formation in renal tissue of rat model

**Fig. 1:**
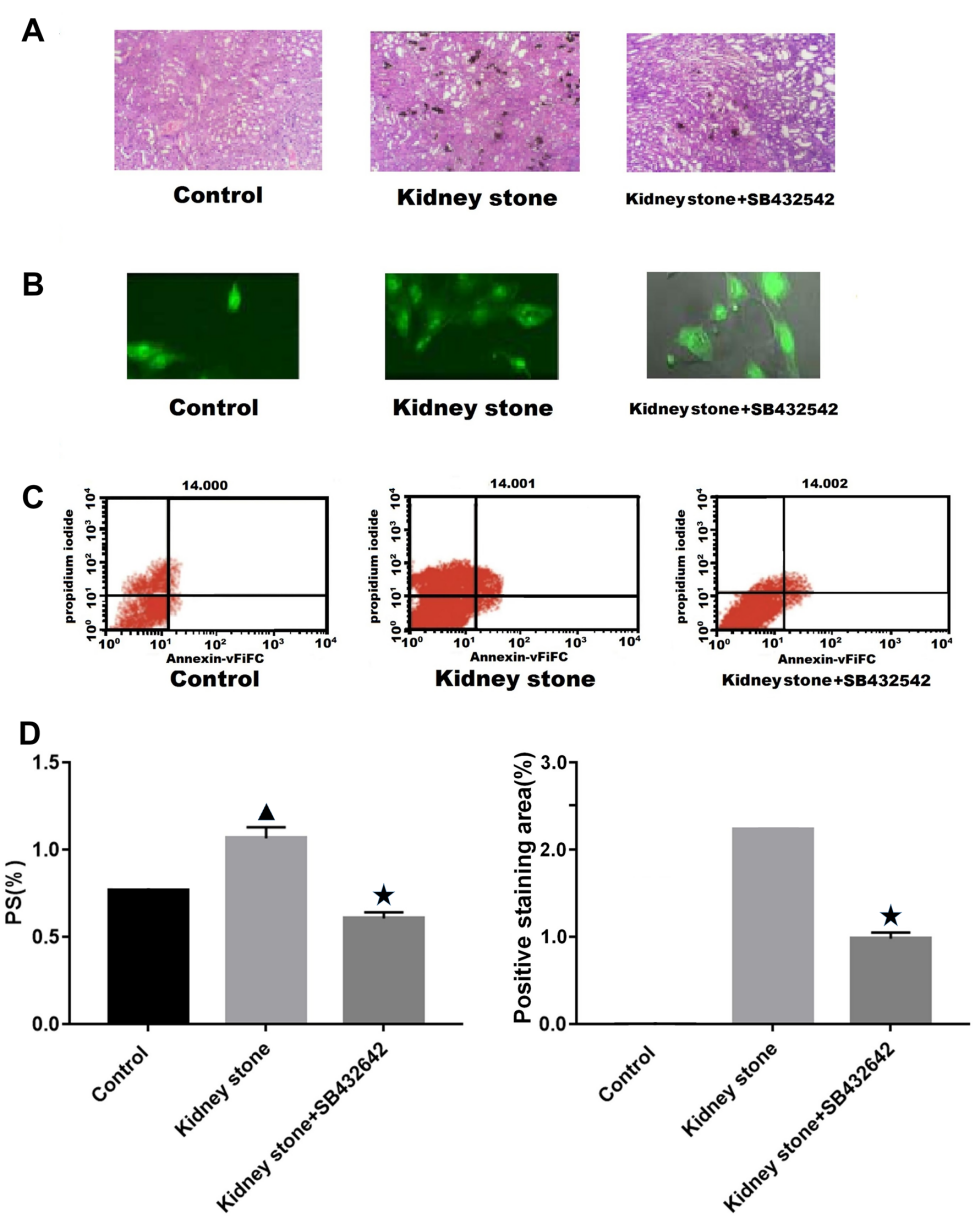
Crystal formation and PS externalization observed in the control, kidney stone, and kidney stone + SB431542 groups. (**A**) Crystal formation in the renal tissue on day 14; arrow indicates microcrystalline structures. (**B**) MDCK cells observed by confocal laser microscopy. (**C**) Flow cytometric plots of eversion rate. (**D**) Eversion rate (%). Triangle indicates *P* < 0.05 vs. control. Asterisk indicates *P* < 0.05 vs. kidney stone group

To establish a rat model of early stage kidney stone formation, ethylene glycol and ammonium chloride were administered to the stomach of rats for 2 weeks, and then each kidney section was examined with a digital microscope. In the control group specimens, normal renal tubular structures were observed without crystal formation on day 14 (Fig. 1A). When the kidney tissue from the kidney stone group was observed by microscopy, the renal tubules appeared dilated with visible microcrystals; occasional accumulation of CaOx crystals was observed in some renal tubules, indicating successful establishment of a rat model of early stage kidney stone formation and the generation of renal tubule cell injury.

#### Presence of PS in the renal tubular cell membrane

Annexin V-FITC, a fluorescent molecule that binds to PS on the cell surface, is widely used to detect PS redistribution. In our study, MDCK cells in the normal control, kidney stone, and kidney stone + SB431542 groups were observed under a confocal laser microscope (Fig. 1B). The PS-eversion rate increased in the kidney stone group compared with that in the control group (*P* < 0.05, Fig. 1C, D), whereas cells in the kidney stone + SB431542 group showed a lower basal level of PS externalization than those in the kidney stone group (*P* < 0.05, Fig. 1C, D).

#### Measurement of TGF-β1 and Smad7 levels

**Fig. 2:**
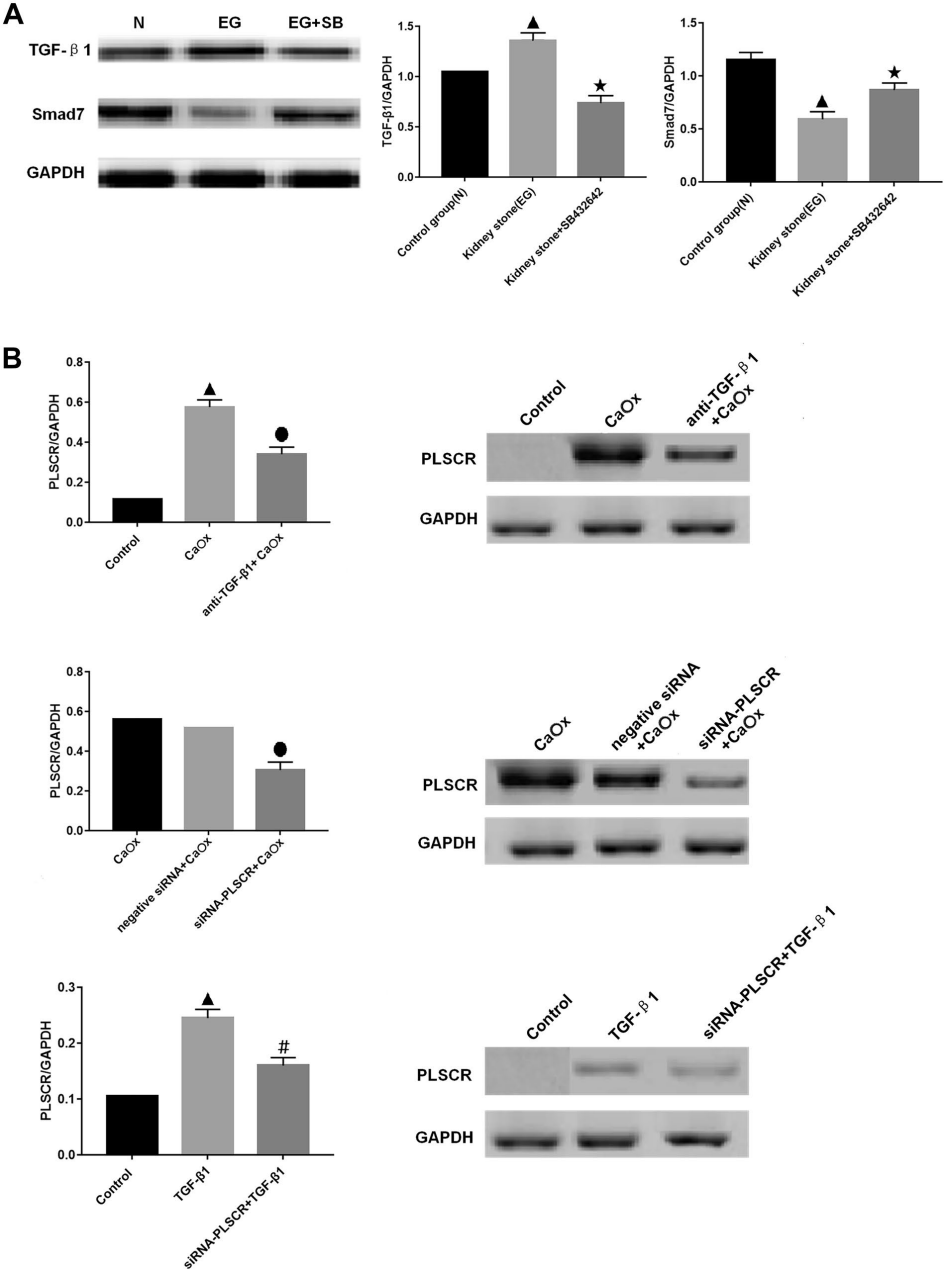
TGF-β1/Smad expression in the control, kidney stone, and kidney stone + SB431542 groups, and PLSCR expression in the renal tubule cell membrane. (**A**) TGF-β1/Smad expression. (**B**) Relative phospholipid scramblase (PLSCR) levels, and representative blot with expression normalized to that of GAPDH. *P* < 0.05. Triangle indicates *P* < 0.05 vs. control. Round dot indicates *P* < 0.05 vs. CaOx group. Pound sign indicates *P* < 0.05 vs. TGF-β1 group

The TGF-β1 level in the kidney stone group was quantified by western blotting. A typical western blot is shown in Fig. 2A together with the densitometric analysis of the TGF-β1 level. Values were normalized to those of the control and expressed as the ratio of TGF-β1 to tubulin. The addition of ethylene glycol and NH_4_Cl significantly increased the TGF-β1 level and decreased the Smad7 level compared with those in the control cells (Fig. 2A); however, treatment with SB431542 + ethylene glycol and NH_4_Cl led to significantly decreased TGF-β1 level and increased Smad7 level compared with those in cells treated with ethylene glycol and NH_4_Cl.

### TGF activates PLSCR activity in the renal tubule cell membrane in vitro

#### PLSCR

The level of PLSCR was evaluated by western blotting. The cells were treated with CaOx for 2 h. The PLSCR level was negligible in control cells but significantly increased in the CaOx group (*P* < 0.05, Fig. 2B). PLSCR overexpression was inhibited following treatment with the anti-TGF-β1 antibody. After the transfection of cells in the TGF-β1 and CaOx groups with PLSCR siRNA, PLSCR expression significantly reduced (*P* < 0.05, Fig. 2B).

#### Impaired NBD-PS inward movement

**Fig. 3:**
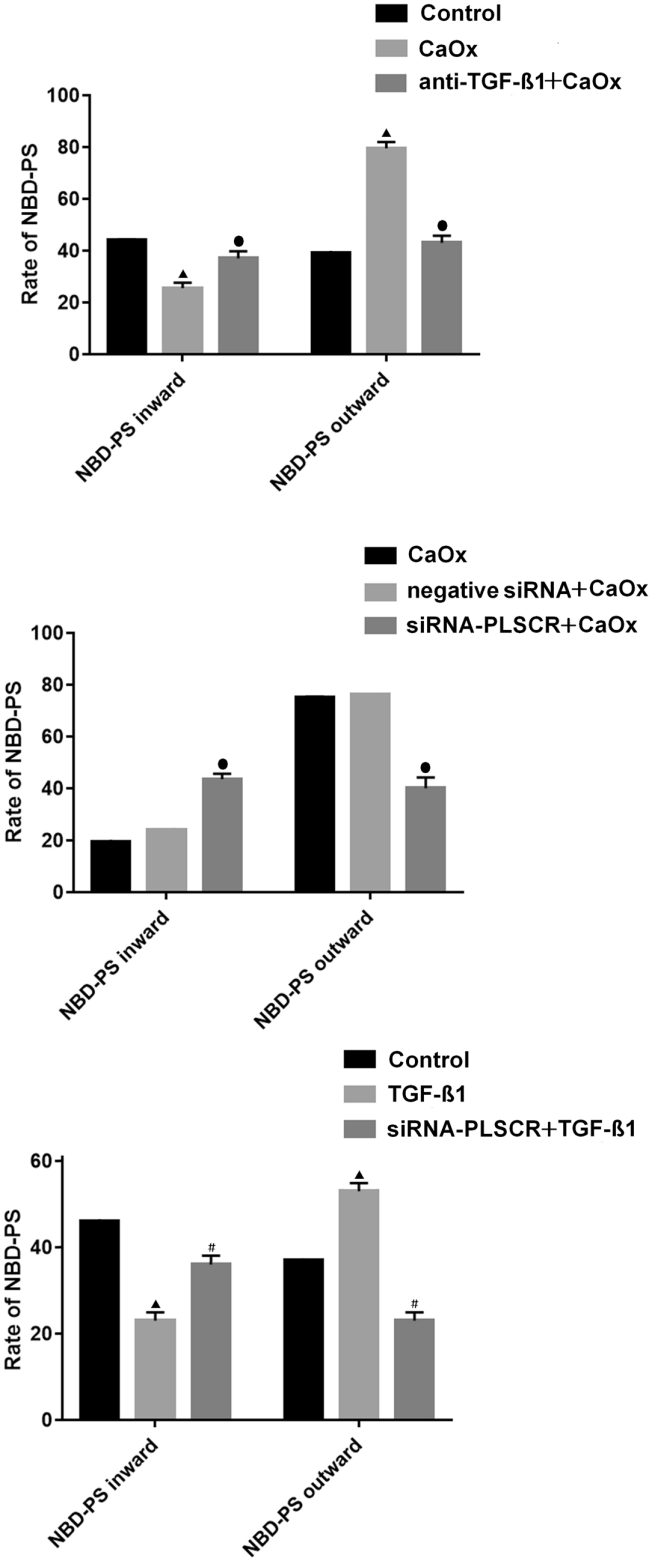
Rate of inward and outward movements of NBD-PS. (**A**) Comparison among the control, oxalate calcium, and anti-TGF-β1 + oxalate calcium groups. (**B**) Comparison among the calcium oxalate, negative + calcium oxalate, and siRNA-PLSCR + calcium oxalate groups. (**C**) Comparison among the control, TGF-β1, and siRNA-PLSCR + TGF-β1 groups. Triangle indicates *P* < 0.05 vs. control. Round dot indicates *P* < 0.05 vs. CaOx group. Pound sign indicates *P* < 0.05 vs. TGF-β1 group

We determined inward and outward movements of PS in the renal tubular epithelial cell membrane to evaluate the PLSCR activity. To assess whether PLSCR influences the movement of PS from the outer leaflet to the inner leaflet of the plasma membrane, we integrated fluorescently labeled PS (NBD-PS) into the outer leaflet of plasma membrane and monitored its internalization (Fig. 3). The amount of NBD-PS internalized by MDCK cells in the control and CaOx groups was approximately 46.2% and 17.8%, respectively. Hence, the rate of PS internalization in the CaOx group cells was significantly lower than that in control group cells (*P* < 0.01, Fig. 3A). However, the level of internalized NBD-PS in the anti-TGF-β1 + CaOx group increased to 34.2%, which was significantly higher than that in the CaOx group (*P* < 0.01, Fig. 3A). After the transfection of cells in the TGF-β1 and CaOx groups with PLSCR siRNA, the level of internalized NBD-PS significantly increased (*P* < 0.05, Fig. 3B, C).

### Enhanced NBD-PS outward movement

To determine whether PS outward transport is affected by oxalate, MDCK cells were intracellularly labeled with NBD-PS, and the extent of its movement to the exoplasmic leaflet was determined. Incubation medium contained bovine serum albumin (1% w/v), which rapidly extracted surface-expressed NBD-PS. Approximately 34.5% of NBD-PS migrated to the outer leaflet in control cells, whereas the rate of outward PS transfer in the CaOx group cells was increased to 75.3% compared with that of the total inner labeled NBD-PS, which was significantly greater than that in control group cells (*P* < 0.01, Fig. 3A). To determine whether TGF-β1 causes PS eversion in renal tubule cell membranes by activating PLSCR, cells in the TGF-β1 and CaOx groups were transfected with PLSCR siRNA. After PLSCR siRNA transfection, the externalization rate was lower than that in the TGF-β1 and CaOx groups (*P* < 0.01 for both, Fig. 3B, C).

## Discussion

To date, animal studies have neither focused on the role of PS in kidney stone formation nor addressed whether the findings of in vitro studies translate to those in animals. To obtain more reliable results, studies using in vitro models are preferable. In our rat model of early stage CaOx stones, the TGF-β1 level and PS-eversion rate in the kidney stone group increased, which is consistent with the results obtained at the cellular level, showing that with increased oxalic acid concentration, a concomitant increase in cell membrane-PS eversion allows crystals to adhere to the cell membrane [28]. Thus, we confirmed that the above cytological studies are suitable for examining the rat model. We found that oxalate itself is injurious to cells and that it may serve as a primary agent to upregulate TGF-β1. Furthermore, the TGF-β1 level and PS externalization were substantially inhibited by SB431542, suggesting that the TGF-β1/Smad signaling pathway facilitates increased PS externalization at the organism level. Progressive activation of NAD(P)H oxidase in renal tubular cells via the induction of TGF-β1, is a potential molecular mechanism of TGF-β1/Smad signaling-induced ROS production [7], whereas ROS mediated accumulation of MRP-1 or BCRP plays a key role in oxalate-induced PS externalization in the renal epithelial cell membrane [23]. Therefore, we conclude that the mechanism of TGF-β1/Smad signaling-induced PS eversion in renal tubular epithelial cells is mediated via oxidative stress caused by ROS.

When LLC-PK1 cells were treated with 1 mmol potassium oxalate (KOx) for 6, 12, 24, or 48 h, Khan et al. did not detect significant apoptotic morphological changes in the cells after 6 h of oxalate treatment. However, after 12 h of exposure, they observed significant apoptotic changes including condensation and margination of nuclear chromatin, DNA fragmentation, and PS migration in the plasma membrane bilayer from inside to the cell surface [8]. In our previous study, we showed that 0.5 mmol oxalate caused a time- and concentration-dependent increase in PS externalization in the MDCK renal epithelial cell line [2]. In this study, 0.5% CaOx was applied to the cells, and the PS-eversion rate increased in the kidney stone group. Although the cell membrane PS turned over in our present study, these cells should not have undergone apoptosis. PS eversion is an important surface marker of apoptosis. Recent studies have shown that apoptosis and PS eversion are two independent events, that is, apoptotic cells do not necessarily show PS eversion and PS-eversion cells are not necessarily apoptotic [29]. It is not appropriate to rely only on PS eversion to determine whether a cell is apoptotic, because studies have reported PS exposure in non-apoptotic cells [30–32].

In this study, negligible level of PLSCR was detected in control group cells, whereas this protein was significantly increased in the CaOx group, indicating overexpression of PLSCR. These results are consistent with the findings of Singireesu et al. regarding the potential toxic effects of DHCL on kidney cells [33]. When cells in the CaOx group were treated with anti-TGF-β1 antibody, the level of PLSCR was significantly lowered, indicating that the activation of PLSCR was mediated by TGF-β1. In addition, we observed enhanced outward movement of NBD-PS in the TGF-β1 group and CaOx group. When renal tubular epithelial cells from these groups were transfected with PLSCR siRNA, the PS internalization rate increased, whereas the externalization rate decreased, indicating that TGF-β1 causes PS externalization in the renal tubule cell membrane by activating PLSCR in the renal tubule cell membrane in vitro. Taken together, oxalate exposure to renal tubule cells induces TGF-β1 expression, resulting in increased ROS production via NAD(P)H oxidase; ROS then alters the activity of PLSCR [9, 10]. Finally, PLSCR leads to PS externalization in the renal tubule cell membrane, as demonstrated in our study.

A previous study suggested that oxalate treatment does not activate PLSCR in MDCK cells in vitro, as PLSCR activation requires increased cytoplasmic Ca^2+^, and oxalate rapidly decreases the intracellular Ca^2+^ concentration in MDCK cells [2]. This hypothesis is supported by the results of the present study. That is, although the previous study demonstrated the effects of oxalic acid, rather than CaOx, on renal tubular epithelial cells, we found that the Ca^2+^ concentration increased in the renal collecting system during the early stage of CaOx stone development in a rat model. As PLSCR is exposed beyond the cell membrane, we believe PLSCR may be activated by Ca^2+^ in the renal collecting system rather than by intracellular Ca^2+^.

Under physiological conditions, an asymmetric distribution of phospholipids in the eukaryotic cell membrane, which requires aminophospholipid translocase (APLT) and PLSCR to play a synergistic role, is necessary for the cell membrane to perform its physiological functions [34]. In this experiment, we considered the ratio of PS eversion in the cell membrane to indicate the activity of PLSCR. Because APLT has been confirmed as one of the factors causing PS eversion, APLT affected the measurement of PLSCR enzyme activity in this experiment. Further clinical studies are required to validate how APLT and PLSCR work together in PS eversion of the renal tubular cell membrane, and to identify other factors controlling PS eversion to better understand the mechanism underlying kidney stone formation.

## Conclusions

CaOx exposure can potentiate the upregulation of TGF-β1, which subsequently activates ROS in renal tubular cells; ROS then strongly activates PLSCR, representing one of the major mechanisms by which TGF-β1-ROS signaling stimulates PS externalization in the renal tubule cell membrane during the early stage of kidney stone development. The results of this study form a basis for further detailed research on the development of therapeutic agents that specifically treat urolithiasis and exert fewer adverse effects.

## Data Availability

The datasets generated during and/or analyzed during the current study are available from the corresponding author on reasonable request.

## Abbreviations

PS: Phosphatidylserine
ROS: Reactive oxygen species
PLSCR: Phospholipid scramblase
TGF-β1: Transforming growth factor-β1
CaOx: Calcium oxalate
EG: Ethylene glycol
